# Supplementary material for: Functional Maturation and Experience‐Dependent Plasticity in Adult‐Born Olfactory Bulb Dopaminergic Neurons
Source: Eur J Neurosci. 2025 Jul 13;62(1):e70188. doi: 10.1111/ejn.70188 (PMC12256165; doi:10.1111/ejn.70188)
Supplement: Supplementary file 1 — Figure S1 BrdU birthdating in DAT‐tdTomato mice. (a) Schematic illustrating the timeline of BrdU injections. P, postnatal day; dpi, days post‐injection. (b) Representative maximum intensity projection images showing BrdU‐positive cells in the glomerular layer of P28 DAT‐tdTomato mice at 7, 14, and 21 dpi. White arrows in the 21 dpi panel indicate cells co‐labelled with BrdU and tdTomato. (c) Mean ± SEM of tdTomato‐positive neurons that were BrdU‐positive at each time‐point (Error bars are omitted when shorter than the symbol height; n = 14,042 cells from N = 12 mice). [file EJN-62-0-s001.pdf]

a)

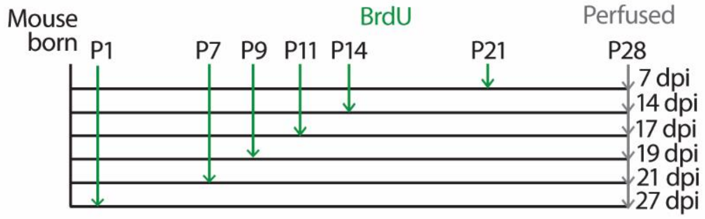
Mouse born
BrdU
Perfused
P1
P7
P9
P11
P14
P21
P28
7 dpi
14 dpi
17 dpi
19 dpi
21 dpi
27 dpi

b)

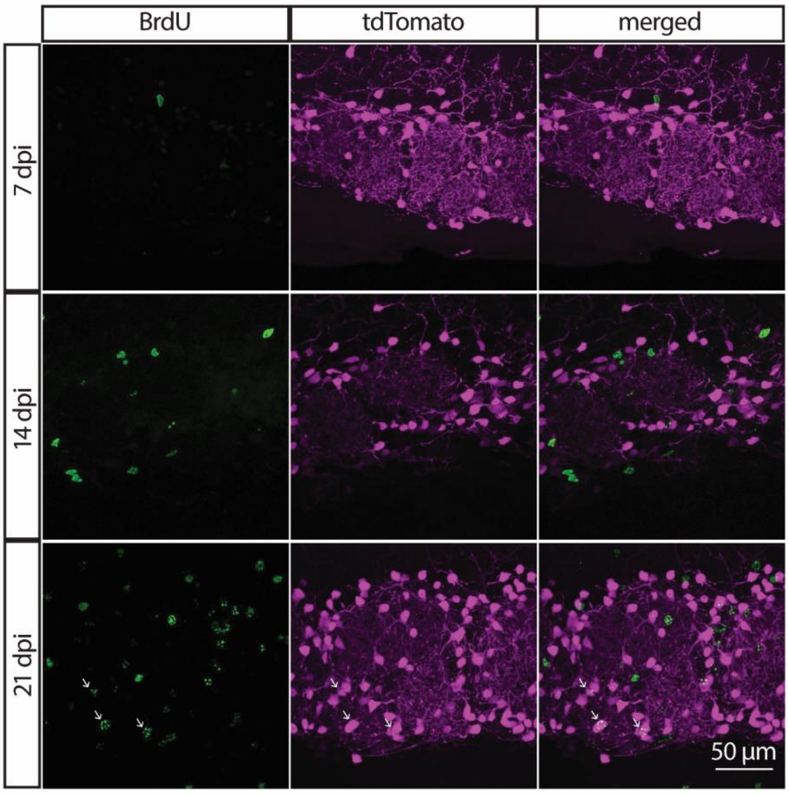
BrdU
tdTomato
merged
7 dpi
14 dpi
21 dpi
50 µm

c)

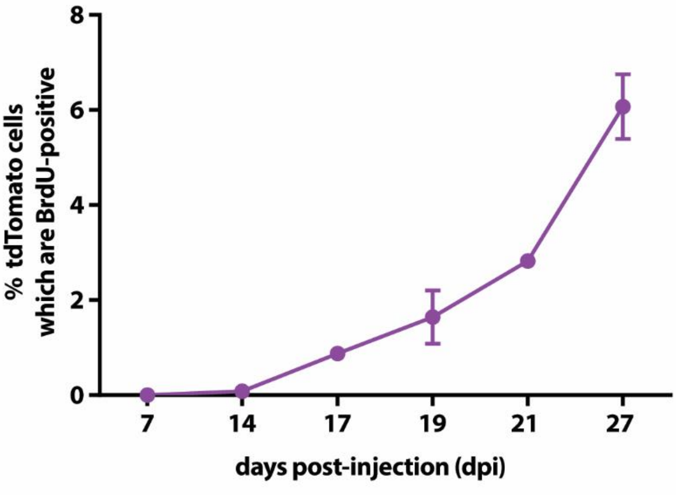
% tdTomato cells which are BrdU-positive
8
6
4
2
0
7
14
17
19
21
27
days post-injection (dpi)

Figure S1. BrdU birthdating in DAT-tdTomato mice. **a)** Schematic illustrating the timeline of BrdU injections. P, postnatal day; dpi, days post-injection. **b)** Representative maximum intensity projection images showing BrdU-positive cells in the glomerular layer of P28 DAT-tdTomato mice at 7, 14, and 21 dpi. White arrows in the 21 dpi panel indicate cells co-labelled with BrdU and tdTomato. **c)** Mean ± SEM of tdTomato-positive neurons that were BrdU-positive at each time-point (Error bars are omitted when shorter than the symbol height; n = 14,042 cells from N = 12 mice).
